# Neglected tropical diseases in Uganda: the prospect and challenge of integrated control

**DOI:** 10.1016/j.pt.2007.08.007

**Published:** 2007-10

**Authors:** Jan H. Kolaczinski, Narcis B. Kabatereine, Ambrose W. Onapa, Richard Ndyomugyenyi, Abbas S.L. Kakembo, Simon Brooker

**Affiliations:** 1 Malaria Consortium Africa, Sturrock Road, PO Box 8045, Kampala, Uganda; 2 Department of Infectious and Tropical Diseases, London School of Hygiene and Tropical Medicine, Keppel Street, London WC1E 7HT, UK; 3 Vector Control Division, Ministry of Health, 15 Bombo Road, Kampala, Uganda

## Abstract

So-called ‘neglected tropical diseases’ (NTDs) are becoming less neglected, with increasing political and financial commitments to their control. These recent developments were preceded by substantial advocacy for integrated control of different NTDs, on the premise that integration is both feasible and cost-effective. Although the approach is intuitively attractive, there are few countrywide experiences to confirm or refute this assertion. Using the example of Uganda, this article reviews the geographical and epidemiological bases for integration and assesses the potential opportunities for, and operational challenges of, integrating existing control activities for several of these diseases under an umbrella vertical programme.

## Potential for integration

Greater emphasis is being given to controlling neglected tropical diseases [NTDs; Neglected tropical diseases, hidden successes, emerging opportunities (http://whqlibdoc.who.int/hq/2006/WHO_CDS_NTD_2006.2_eng.pdf)]. The term ‘NTDs’ is used because they exclusively affect the poor and marginalized in low-income countries and, until recently, received little or no advocacy or funding. The most important African NTDs are shown in Table 1. Although these diseases are thought to kill up to 500 000 people per year [1], mortality figures alone do not capture the main impact of NTDs on public health, which largely arises from chronic disability and morbidity [2]. In an effort to control or eliminate this disease burden, several global vertical initiatives have been established [3]. Since 2004, there has been greater advocacy for the logistical and economic benefits of integrated control of NTDs, whereby different treatment strategies are bundled together [4–6]. Integration can also involve another aspect: linking intervention packages with ongoing healthcare delivery [7].

Small-scale efforts to integrate vertical NTD programmes have been undertaken in several African countries. For example, in Nigeria, integrated distribution of anthelmintic treatments combined with insecticide-treated nets (ITNs) by community-based volunteers resulted in increased uptake of ITNs without an adverse effect on the coverage of mass drug administration (MDA) [8]. To help support national programmes, the World Health Organization (WHO) has recently published guidelines on integrated helminthiasis control, which have been designed to deal with drugs and their coordinated use in all epidemiological situations, including those in which there is limited geographical overlap [Preventive chemotherapy in human helminthiasis (http://whqlibdoc.who.int/publications/2006/9241547103_eng.pdf)]. In addition, the Global Network for Neglected Tropical Disease Control was launched in October 2006, with the aim of providing advocacy and coordinating the efforts of NTD-control partners [9]. However, although the concept of integration is logistically and economically appealing, experience at the country level is surprisingly limited.

Similar to many other developing countries, Uganda is affected by a high burden of NTDs: visceral leishmaniasis (VL; kala-azar) [10], human African trypanosomiasis (HAT) [11], trachoma [12], Buruli ulcer [13], soil-transmitted helminths (STHs) [14], schistosomiasis owing to *Schistosoma mansoni* [15], lymphatic filariasis (LF) [16] and onchocerciasis [17] (Table 2). Dracunculiasis and leprosy have recently been eliminated from the country [18]. Uganda provides a useful insight into the control of NTDs because it is one of the few African countries that has undertaken nationwide assessments for several NTDs [15–17] and already piloted integrated control [19]. It also implements a broader integrated health package through the Child Health Days (CHDs) instigated by the Ministry of Health (MoH) and is one of five African ‘fast-track’ countries that receives support from the US Agency for International Development (USAID), to develop an integrated NTD control programme [RTI launches integrated program to address neglected tropical diseases (http://www.rti.org/newsroom)]. Implementation of such a package necessitates careful consideration of several issues, including the geography, epidemiology and ecology of different NTDs, in addition to the advantages and disadvantages of existing control strategies.

## Geography of integrated control

Understanding which geographical areas require intervention is fundamental for cost-effective disease control. NTDs in Uganda have been mapped using a variety of survey methodologies. The distribution of onchoceriasis has been estimated using the rapid epidemiological mapping of onchoceriasis (REMO) method [20], enabling communities to be classified into three categories: priority areas requiring community-directed drug treatment with ivermectin (CDTI); areas not requiring treatment; and possible endemic areas requiring further investigation [17]. Rapid mapping of LF included school surveys using immunochromatographic antigenic detection cards and the application of geostatistical methods to create a nationwide estimation of the prevalence of LF [16]. The distributions of schistosomiasis and STH infections were defined according to nationwide parasitological surveys [14,15]. More recently, rapid mapping of schistosomiasis used lot quality-assurance sampling (LQAS) to finely target control [21]. LQAS has also been used to estimate the prevalence of *Trypanosoma brucei gambiense* trypanosomiasis in northern Uganda, enabling communities to be ranked according to prevalence categories [22]. Elsewhere, distributions of HAT have been assessed using expensive case detection by passive or population mass screening: *T. b. gambiense* occurs in northwestern Uganda, whereas *Trypanosoma brucei rhodesiense* has traditionally occurred in southeastern areas [23]. These two foci are currently geographically separated but are becoming worryingly close [11,24]. The endemicity of VL has, so far, been defined only on the basis of passive case-detection data, which suggest that the disease is restricted to Pokot county, a semiarid lowland area in the Nakapiripirit district [10]; however, there are concerns that VL might be endemic over a larger area. Trachoma is thought to be endemic in at least 26 districts, putting ∼7 million people at risk. A nationwide survey is planned, to provide detailed data on the distribution and burden of trachoma.

**Figure 1:**
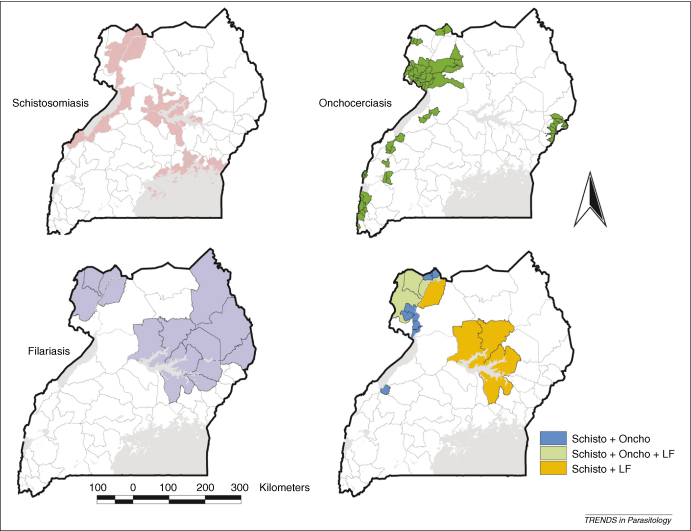
Areas of Uganda endemic or coendemic for NTDs that are controlled using MDA of preventative chemotherapy. Areas shown in red are endemic for schistosomiasis, light green areas are endemic for onchocerciasis, yellow areas are endemic for VL and light blue areas are endemic for LF. Dark blue areas indicate counties (administrative areas below district level) coendemic for schistosomiasis and onchocerciasis, orange areas are districts in which schistosomiasis and LF are coendemic and dark green areas are districts in which schistosomiasis, LF and onchocerciasis are present. STHs are endemic throughout Uganda.

On the basis of these geographical assessments, it is possible to qualitatively define the codistribution of different NTDs (Figure 1). Existing data indicate that onchocerciasis, schistosomiasis and LF are coendemic in ten districts of northwestern Uganda, putting >500 000 people at risk of coacquiring them. LF is coendemic with schistosomiasis in at least 19 districts and with onchocerciasis in at least 13 districts. Further surveys are required to confirm whether coendemicity applies to whole districts or is more localized.

## Epidemiology and ecology of integrated control

Control of different NTDs must be based on a detailed understanding of their epidemiologies and modes of parasite transmission. The target age-groups might differ between NTDs [4]. The prevalence and intensity of schistosomiasis and STHs (except hookworm) are greatest among school-age children or young adults and decrease throughout adulthood [15,25], whereas for LF and hookworm age-specific prevalence rises throughout childhood and attains a stable asymptote, or rises marginally, in adulthood [26–28]. Epidemiological patterns of onchocerciasis vary markedly between geographical zones [29]; in Uganda, the prevalence of infection increases throughout childhood and reaches a plateau at 20 years, whereas the occurrence of nodules and onchocercal dermatitis increases throughout childhood and adulthood [30,31]. Thus, school-age children are the natural targets for population-based treatment of STHs and schistosomiasis, whereas communitywide treatment is warranted for LF and onchocerciasis.

LF and onchocerciasis are vector-borne diseases, transmitted by several genera of mosquitoes and blackflies of the *Simulium* genus, respectively. Vector control has been highly effective in the control of onchocerciasis [32], for which the stated goal is interruption of transmission, and might potentially have a significant role in elimination of LF [33]. In both cases, communities within whole districts should be targeted with interventions [34,35]. Transmission of STHs and schistosomiasis depends on contamination of soil and snail-infested water with human faeces and urine, hindering elimination in settings with inadequate water supply and sanitation. Consequently, the goal of schistosomiasis and STH control is the reduction of morbidity, hence interventions typically target age-groups with the greatest morbidity, namely school-age children and young adults in high-prevalence communities or subdistricts [36,37]. These different treatment goals and intervention units require consideration in the design of integrated treatment programmes for NTDs.

HAT is transmitted by tsetse flies and occurs more often in adults [38], whereas VL is transmitted by sandflies and, at least in Uganda, is most common in children and teenagers [10]. Vector control can make an important contribution to reducing the burden of both diseases [39–41], but it is rarely implemented because of a lack of financial resources. Treatment is lengthy, expensive and relatively toxic. Development of new drugs and adequate diagnostic tools has been slow [42,43], although a reliable rapid diagnostic test for VL is now available [44].

## Current control of neglected tropical diseases in Uganda

The control of most NTDs is the mandate of the Vector Control Division (VCD) of the Ugandan MoH. The VCD was established in the early 1920s and led national vector-borne disease control until the 1970s, when it virtually collapsed during military rule, only being rehabilitated in 1994 [45].

The longest-running control programme in the VCD is the national onchocerciasis control programme, established in 1992. Since the mid-1990s, it has been supported by the Carter Centre's Global 2000 River Blindness Programme, Sight Savers International, the Gesellschaft für Technische Zusammenarbeit and the African Programme for Onchocerciasis Control. Intervention consists of annual CDTI, supplemented by vector control in isolated foci of *Simulium neavei* [46,47]. To date, geographical treatment coverage has reached 100% and therapeutic coverage remains stable at 80%. Large-scale vector control is unfeasible because the breeding sites are too widespread or inaccessible and extend into politically unstable countries, such as the Democratic Republic of Congo.

The cornerstone of LF control is annual MDA of a single dose of ivermectin and albendazole, provided to the entire ‘at-risk’ population in targeted districts. The first MDA for LF was carried out at the end of 2002 in two districts with a total population of 1 million people, reaching ∼75% coverage. Scaling up the programme to cover eight adjacent districts, planned for 2003, was delayed because of insecurity and insufficient operational funds. In 2004, MDA was carried out in five districts, with a total population of >2 million, and, in 2005, the programme was extended to cover ten districts, with a total population of 4.9 million. In 2006 no distribution took place, owing to a lack of funds for drug delivery. MDA is carried out in schools and communities by trained teachers and community drug distributors (CDDs), respectively, with most districts having reached at least 65% coverage. It is increasingly appreciated that the use of ivermectin and albendazole in MDA for the elimination of LF has ancillary benefits against onchocerciasis and STHs [4].

For the combined control of schistosomiasis and STH infections, a national programme was established in 2003 [48], with support from the Schistosomiasis Control Initiative. The programme is managed centrally by the VCD, but it is implemented by district health teams. MDA of praziquantel and albendazole is provided to all school children in target subcounties (at the subdistrict level) and the whole community in areas where prevalence of infection exceeds 50%. Treatment is carried out by teachers and CDDs in schools and communities, respectively [49].

HAT control activities, consisting of the mass treatment of livestock using trypanocides and vector control, were implemented in parts of the Soroti district between January 2000 and December 2003. However, a survey conducted in 2004 of Soroti markets showed a high prevalence of *T. b. rhodesiense* in cattle bought from areas in southeast Uganda with endemic sleeping sickness. This showed that control activities have been largely ineffective, and the trade and resultant movement of animals infected with trypanosomes continues [11]. Currently, no control is undertaken against VL, Buruli ulcer or trachoma.

## Progress and prospects of integrated control

The feasibility study of integrating treatment for onchocerciasis with schistosomiasis and STH infections showed that the treatment coverage of ivermectin, praziquantel and mebendazole were increased using the integrated approach [19]. An identified disadvantage was that supplies of praziquantel and mebendazole ran out more frequently, because treatments were being administered to nontarget groups. The investigators suggested that the CDDs might have thought that they or their immediate relatives had schistosomiasis and/or STH infections, and thus treated themselves or their family before treating the targeted high-risk groups in the neighbourhood. Despite the promising results, integration has not been put into practice to date, although financial support from the USAID aims to expand integrated delivery of anthelmintic treatment from 2007.

STH control also forms one of the components of the CHDs in Uganda, which take place twice each year (in April and October). CHDs are a period of accelerated routine maternal and child health interventions, delivered at all static health units and through outreach in communities and schools. The package of interventions includes vitamin A supplementation, childhood vaccination and promotion of hygiene at home and school. Implementation is through a multidisciplinary team of health workers, community members (including CDDs), vaccinators and mobilizers. The provision of annual albendazole treatment by this integrated approach improves the nutritional status of young children [50].

The strategy proposed for integrated control of LF, onchocerciasis, schistosomiasis, STHs and trachoma, to be supported by the USAID, focuses on the integration of individual drug-delivery activities under an umbrella programme, to provide simultaneous, or almost simultaneous, population-based treatment. Only four drugs – albendazole, ivermectin, praziquantel and azithromycin – are used to control seven major NTDs – schistosomiasis, hookworm, trichuriasis, ascariasis, trachoma, LF and onchocerciasis. These NTDs exhibit considerable geographical overlap [1], at least if viewed at the country level [6]. It is thus thought that a single structure, such as CDTI, CHDs or the National Malaria Control Programme, could be readily used to deliver more than one treatment. Because the structures are already in place, this would, in theory, only slightly increase costs if a component is added or reduce costs if two structures are merged, while considerably expanding coverage [4,6,51,52]. In Uganda, however, there is a limited geographical overlap between the different NTDs (Figure 1), necessitating a more geographically targeted approach. Furthermore, the structural changes required to deliver an integrated package are still being undertaken. In the interim, it is already planned that the LF programme will provide ivermectin in April each year and the onchocerciasis control programme will provide ivermectin in October each year. In areas coendemic for LF and onchocerciasis, such an approach has the potential to eliminate both diseases [53,54].

In addition to differences in delivery structure and target geographical areas, the frequency of drug administration varies between control programmes. Treatment for STHs is recommended every 6–12 months, whereas treatment for onchocerciasis and LF is recommended annually. The actual frequency and number of rounds of ivermectin treatment required to interrupt transmission of LF or onchocerciasis are unknown [29]. Although schistosomiasis treatment using praziquantel is currently provided annually, longer treatment intervals might become justified as infection levels decrease. Coordinating these different treatment intervals represents a challenge for integration.

Treatment regimens for HAT and VL are too toxic and lengthy to be delivered outside a health facility [23,42,55] and are thus unsuitable for inclusion in this new integrated approach. A threat exists, therefore, that control of HAT, VL and other NTDs will continue to be neglected, as attention is focused on diseases that have a population-based chemotherapy strategy. Recent NTD advocacy has contributed to the allocation of funds for the development of a new generation of control tools (drugs, diagnostics and vaccines) for VL and HAT, in addition to other NTDs [e.g. the Drugs for Neglected Diseases *initiative* (http://www.dndi.org) and Sabin Vaccine Institute (http://www.sabin.org)], but has had little impact on the allocation of funds to deliver existing HAT and VL control tools. Until new tools become available, control with existing, although imperfect, tools must be intensified [42,56].

In addition to integrating treatment, there is considerable potential for integrated vector control for several NTDs, which receives little mention. In Uganda, the same mosquito species transmit both LF and malaria in the same districts [26]. Increasing the coverage with long-lasting ITNs (LLINs), as part of the malaria control efforts, is thus likely to impact on LF vector densities and transmission [57,58] and merits further investigation [Roles of vector control and xenomonitoring in LF elimination (http://whqlibdoc.who.int/hq/2002/WHO_CDS_CPE_PVC_2002.3.pdf)]. Use of LLINs is also likely to provide personal protection against sandfly vectors of VL [59]. Because VL and malaria are coendemic in Uganda, scaling up of LLIN coverage in the VL endemic area would be a good investment in health.

## Challenges for integrated control

Approaches for integrated control are still being developed and best practice will only emerge after experience of actual implementation. Opportunities for implementation on a national scale are now being created through the USAID funding. In designing and implementing country programmes, several operational challenges exist and integrated control might not be as straightforward and cost-effective as it is portrayed [Strengthening the potential of health systems in rural Africa (http://bmj.bmjjournals.com/cgi/eletters/328/7448/1129)] [60]. Potential shortcomings include an increased bureaucratic burden, leading to reduced effectiveness of health services [Health services are badly needed to control malaria (and other diseases) (http://bmj.bmjjournals.com/cgi/eletters/328/7448/1129)] [7]. Also, as the number of interventions increases, the activities of the CDDs resemble those of a full-time job and the CDDs cannot attend to other activities that generate income. The increased workload might prove detrimental to their performance in any one activity, as already documented for the onchocerciasis control programme [61], and lead to demands for incentives in compensation for the work [62]. Whether, and to what extent, the capacity of CDDs in Uganda is underused requires further investigation, but it is already apparent that all programmes that heavily draw on them experience increasing demands for incentives [63,64]. These demands could, potentially, be overcome by increasing the number of CDDs so that the workload of each individual is reduced. However, to increase the pool of CDDs, more funding would be needed for training, health education, monitoring and supervision.

The current model of integrated MDA differs from the more common understanding of integration as ‘a process where disease control activities are functionally merged or tightly coordinated with multifunctional health care delivery’ [7]. Therefore, another challenge is the possibility that linking vertical control programmes might promote the development of a parallel health-delivery system, with separate funding, drugs, delivery channels and staff [The challenge of global health (http://www.foreignaffairs.org/20070101faessay86103/laurie-garrett/the-challenge-of-global-health.html)]. Ideally, drugs should be distributed from the centre to health facilities, which then distribute the drugs to CDDs and schools, as part of their outreach activities. Health workers should also be involved in training, monitoring and supervision. If the programme is to be sustainable in the long term and not reliant on continual donor support, it is essential that interventions are delivered through existing MoH staff and funded at national and local levels.

Further challenges are the harmonization of information, education and communication (IEC) messages and their effective delivery. To date, social mobilization and sensitization of target communities have often been inadequate, because resources for activities, such as surveys of knowledge, attitude and practice, development of IEC materials and community meetings, were limited. Furthermore, for both the STH and schistosomiasis programme and the onchocerciasis control programme, communities were sometimes not involved in the selection of their CDDs. In these cases, communities were reluctant to participate in control activities and CDDs were more likely to ‘drop out’ [63]. These experiences show that resources are urgently needed to improve on development, implementation and evaluation of the health-education component of each programme and communities must be empowered to select their own health workers. An integrated approach will face the same challenges.

The safety and efficacy of certain drug combinations is also unknown [65]. Combinations currently approved by the WHO [Preventive chemotherapy in human helminthiasis (http://whqlibdoc.who.int/publications/2006/9241547103_eng.pdf)] are shown in Table 3. Studies of coadministration of both ivermectin, albendazole and praziquantel and anthelmintic treatments and zithromax are required [60]. Implementation of integrated chemotherapy that has unknown potential side effects must be accompanied by vigorous pharmacovigilance. A general pharmacovigilance system is currently being put in place in Uganda, but its implementation already poses numerous practical challenges [66]. These, and the need for additional training, monitoring and supervision of health workers, should limit implementation of an integrated package to a pilot area and be supported by a strong operational research component designed to yield the necessary evidence on safety, effectiveness and operational constraints [60].

Finally, monitoring and evaluation activities must be carefully designed and implemented, to answer important operational questions and modify and support control packages, as necessary. Guidance on the epidemiological aspects of evaluating helminth control programmes is already available [67] and a WHO manual on evaluating integrated control is currently being developed. However, evaluation of the health benefits of an integrated control package represents a major challenge.

## Concluding remarks

The success of integrated control depends on a clear understanding of the distribution and epidemiology of the diseases to be targeted. In most countries, this information is incomplete, requiring detailed surveys to establish areas of coendemicity and formulate MDA packages accordingly. With the move towards integrated control, there is a need to broaden the scope of research, including studies of the effectiveness and cost-effectiveness of integrated control of NTDs compared with existing control programmes. There is also a need to evaluate the impact of integration on existing health systems, including the quality of health care and staffing levels. Efforts to implement integrated control must be accompanied by investment in, and strengthening of, healthcare systems and human resources, because these are prerequisites for the success of global health initiatives [68].

It is hoped that other health-sector donors will soon follow the example of the USAID and start to support the unmet needs for control of NTDs. Resources are urgently required to establish an evidence base for integrated control and curb the burden of diseases that cannot be controlled through MDA. Case management of these diseases must become a functioning component of the existing healthcare system [56]. Obvious gaps in the Ugandan context are HAT and VL, which will not benefit from integrated control as it is currently planned.

## Figures and Tables

**Table 1:** NTDs and their control in Africa

Disease	Aetiologic agent	Distribution	Control strategy	Drugs	International programmes
***Helminth***					
STHs	*Ascaris lumbricoides*	*A. lumbricoides* and *T. trichiura* restricted to equatorial regions; hookworm is widespread	Annual mass treatment of schoolchildren and whole communities in high-prevalence areas	Benzimidazole anthelmintic treatments, albendazole and mebendazole	Mebendazole donation initiative supported by Johnson and Johnson (http://www.taskforce.org/mebendazole)
	*Trichuris trichiura*				
	Hookworm				
Schistosomiasis (bilharziasis)	*Schistosoma haematobium*	Africa-wide	Annual mass treatment of schoolchildren and whole communities in high-prevalence areas	Praziquantel	Schistosomiasis control initiative (http://www.schisto.org)
	*S. mansoni*				
LF (elephantiasis)	*Wuchereria bancrofti*	Endemic in 39 African countries	Annual MDA to treat the entire population for a (currently undefined) long period, to interrupt transmission	Albendazole and ivermectin	Global Alliance for the Elimination of Lymphatic Filariasis (http://www.filariasis.org)
Onchocerciasis (river blindness)	*Onchocerca volvulus*	Endemic in 30 African countries	Vector control through spraying of larvicides and annual CDTI	Ivermectin	African Programme for Onchocerciasis Control (http://www.Apoc.bf/en/)
Dracunculiasis (Guinea worm)	*Dracunculus medinensis*	Eliminated as a public health problem	Active case detection, provision of a water supply and use of cloth filters		Guinea worm eradication programme (http://www.cartercenter.org/health/guinea_worm/index.html)

***Protozoan***					
Cutaneous leishmaniasis	*Leishmania tropica*	Scattered foci throughout Africa	Case detection and treatment. Personal protection through use of mosquito nets	Pentavalent antimonial treatments; the second-line drug is amphotericin	
	*Leishmania major*				
	*Leishmania infantum*				
VL (kala-azar)	*Leishmania donovani*	Scattered foci in the Horn of Africa, Sudan, Ethiopia, Somalia, Kenya and Uganda	Case detection and treatment. Personal protection through use of mosquito nets	Pentavalent antimonial treatments; the second-line drug is amphotericin	Drugs for Neglected Diseases initiative (http://www.dndi.org/)
HAT	*T. b. gambiense*	Endemic in 37 African countries	Case detection and treatment. Vector control through spraying, traps and targets	*T. b. rhodesiense*: suramin or melarsoprol in early- or late-stage disease, respectively	Programme against African trypanosomiasis (http://www.fao.org/ag/againfo/programmes/en/paat/home.html)
	*T. b. rhodesiense*			*T. b. gambiense*: pentamidine or suramin for early- or late-stage disease, respectively. The alternative for melarsoprol-refractory late-stage *T. b. gambiense* treatment is eflornithine	

***Bacterial***					
Trachoma	*Chlamydia trachomitis*	Widespread throughout the continent	Surgery, antibiotic therapy, facial cleanliness and environmental improvement (SAFE) strategy	Zithromax	International trachoma initiative (http://www.trachoma.org)
Buruli ulcer	*Mycobacterium ulcerans*	Reported cases from eight west African countries, seven central Africa countries, Malawi and Uganda	Case detection, treatment and surgery	Rifampicin and streptomycin or amikacin	
Leprosy	*Mycobacterium leprae*	Close to elimination (defined as prevalence of <1 case per 10 000 population), although pockets of high endemicity remain in several areas of Angola, the Central African Republic, the Democratic Republic of Congo, Madagascar and Tanzania	Multidrug therapy	Dapsone and rifampicin	

**Table 2:** NTDs in Uganda

Disease	Distribution^a^	Nationwide burden	Refs
*A. lumbricoides* and *T. trichiura*	Unevenly distributed; the highest prevalence is in southwest Uganda	Average prevalence of <10%, but >50% in southwest Uganda	[14]
Hookworm	Throughout Uganda (the prevalence is lower in the northeast)	Prevalence of >50%	[14]
Schistosomiasis	In 30 districts, particularly near the shores of lakes Albert and Victoria and along the Albert Nile	About 4 million cases; 16.7 million are at risk	[15]
LF	North of the Victoria Nile and in west Uganda	Prevalence of circulating filarial antigens in schoolchildren is 0.4–30.7%; 13.9 million are at risk	[16]
Onchocerciasis	In 27 districts; highly endemic in the west Nile region, central shores of lake Albert, Mount Elgon and foci in southwest Uganda	Greater than 2 million at risk; 1.36 million infected	[17]
Dracunculiasis	Eliminated as a public health problem	Eliminated	[18]
VL	Pokot county and the Nakapiripirit district (northeast Uganda)	Unknown; >600 cases treated per year, of which 70% are from Kenya	[10]
HAT	Northwest Uganda, predominantly in the Adjumani, Moyo, Arua and Yumbe districts	In 2005, 267 cases were reported	
	Southeast and east Uganda	In 2005, 479 cases were reported	[11,24]
Trachoma	In 15 districts (according to HMIS records); a nationwide survey is planned	Unknown	[12]
Buruli ulcer	Unknown	Unknown	
Leprosy	Eliminated as a public health problem	In 2004, 2.5 new cases per 100 000 population	b

a The number of districts quoted here and elsewhere in the document refers to the number prior to recent administrative changes that have divided some of the previous districts.

b GLRA/NTPL. *Leprosy Status Report 2004*. German Leprosy Relief Association/National TB and Leprosy Programme. Wandegeya, Kampala, Uganda, 2004.

**Table 3:** Summary of approved preventative schedules for helminthic diseases

Disease	Treatment
LF	Treat the entire population at risk using ALB and DEC or ALB and IVN
LF and onchocerciasis	Treat the entire population at risk using ALB and IVN
LF and schistosomiasis	Round 1: treat the entire population at risk using ALB and DEC or ALB and IVN
	Round 2 (at least 1 week after Round 1): treat school-age children and adults at risk using PZQ
LF and STHs	Round 1: treat the entire population at risk using ALB and DEC or ALB and IVN
	Round 2 (after 6 months): if the prevalence of STH is ≥50%, treat school-age children using ALB or MEB
LF, onchocerciasis and schistosomiasis	Round 1: treat the entire population at risk using ALB and IVN
	Round 2 (at least 1 week after Round 1): treat school-age children and adults at risk using PZQ
LF, onchocerciasis and STHs	Round 1: treat the entire population at risk using ALB and IVN
	Round 2 (after 6 months): if the prevalence of STH is ≥50%, treat school-age children using ALB or MEB
Onchocerciasis	Treat the entire population at risk in meso- and hyperendemic communities using IVN
Onchocerciasis and schistosomiasis	Round 1: treat the entire population at risk in meso- and hyperendemic communities using IVN
	Round 2 (at least 1 week after Round 1): treat school-age children and adults at risk using PZQ
Onchocerciasis and STHs	Round 1: ALB (treat school-age children) and IVN (treat the entire population at risk in meso- and hyperendemic communities)
	Round 2 (after 6 months): if the prevalence of STH is ≥50%, treat school-age children using ALB or MEB
Schistosomiasis	Treat school-age children and adults at risk using PZQ
Schistosomiasis and STHs	Round 1: ALB or MEB (treat school-age children) and PZQ (treat school-age children and adults considered at risk)
	Round 2 (after 6 months): if the prevalence of STH is ≥50%, treat school-age children using ALB or MEB
STHs	Round 1: treat school-age children using ALB or MEB
	Round 2 (after 6 months): if the prevalence of STH is ≥50%, treat school-age children using ALB or MEB

ALB, albendazole; DEC, diethylcarbamazine; IVN, ivermectin; MEB, mebendazole; PZQ, praziquantel; LF, lymphatic filariasis; STH, soil-transmitted helminths.
